# Post-Stroke Mortality, Stroke Severity, and Preadmission Antipsychotic Medicine Use – A Population-Based Cohort Study

**DOI:** 10.1371/journal.pone.0084103

**Published:** 2014-01-08

**Authors:** Anders Prior, Thomas Munk Laursen, Karen Kjær Larsen, Søren Paaske Johnsen, Jakob Christensen, Grethe Andersen, Mogens Vestergaard

**Affiliations:** 1 Research Unit for General Practice, Department of Public Health, Aarhus University, Aarhus, Denmark; 2 Section for General Practice, Department of Public Health, Aarhus University, Aarhus, Denmark; 3 National Centre for Register-based Research, Aarhus University, Aarhus, Denmark; 4 Department of Clinical Epidemiology, Aarhus University Hospital, Aarhus, Denmark; 5 Department of Neurology, Aarhus University Hospital, Aarhus, Denmark; Kaohsiung Chang Gung Memorial Hospital, Taiwan

## Abstract

**Background and Purpose:**

It has been suggested that antipsychotic medication may be neuroprotective and may reduce post-stroke mortality, but studies are few and ambiguous. We aimed to investigate the post-stroke effects of preadmission antipsychotic use.

**Methods:**

We conducted a nationwide, population-based cohort study of 81,143 persons admitted with stroke in Denmark from 2003–2010. Using Danish health care databases, we extracted data on preadmission use of antipsychotics and confounding factors. We examined the association between current, former, and never use of antipsychotics and stroke severity, length of hospital stay, and 30-day post-stroke mortality using logistic regression analysis, survival analysis, and propensity score matching.

**Results:**

Current users of antipsychotics had a higher risk of severe or very severe stroke on The Scandinavian Stroke Scale than never users of antipsychotics (adjusted odds ratios, 1.43; 95% CI, 1.29–1.58). Current users were less likely to be discharged from hospital within 30 days of admission than never users (probability of non-discharge, 27.0% vs. 21.9%). Antipsychotics was associated with an increased 30-day post-stroke mortality among current users (adjusted mortality rate ratios, 1.42; 95% CI, 1.29–1.55), but not among former users (adjusted mortality rate ratios, 1.05; 95% CI, 0.98–1.14).

**Conclusions:**

Preadmission use of antipsychotics was associated with a higher risk of severe stroke, a longer duration of hospital stay, and a higher post-stroke mortality, even after adjustment for known confounders. Antipsychotics play an important role in the treatment of many psychiatric conditions, but our findings do not support the hypothesis that they reduce stroke severity or post-stroke mortality.

## Introduction

It has recently been suggested that antipsychotic medication provides a neuroprotective effect and reduces post-stroke mortality in humans [1]. Thus, a population-based cohort study from Taiwan found that patients with schizophrenia had a lower 90-day post-stroke mortality than patients without schizophrenia; however, the lower mortality was found only among patients who had received antipsychotics before their stroke.

Furthermore, animal studies of acute stroke have indicated that antipsychotic medication may have neuroprotective effects that are related to their antiserotonergic and antidopaminergic mechanisms of action leading to reduced effects of glutamate [2]–[5]. A reduced effect of glutamate may theoretically decrease excitotoxicity and increase neuronal survival [6]–[10], but current results are ambiguous [11]–[13].

Antipsychotics are used in a broad range of diseases other than schizophrenia. Such diseases include psychotic and affective disorders, personality and anxiety disorders, and agitation in dementia [14], [15]. Long-term use of antipsychotics reduces overall mortality in schizophrenia [16], but not in dementia [17]. It has been recommended not to use antipsychotics after stroke because a small study showed impaired stroke recovery in patients treated with antipsychotics [18], [19]. However, more detailed and more comprehensive data on the possible clinical impact of antipsychotics on the clinical outcome of patients with stroke are warranted. We hypothesized that antipsychotics might improve patient outcomes after stroke.

In the present nationwide population-based cohort study, we compared post-stroke mortality, severity of stroke, and length of hospital stay in patients with and without preadmission use of antipsychotics.

## Materials and Methods

### Study population

The study population included all patients over 18 years admitted to Danish hospitals with acute stroke between 1 January 2003 and 31 December 2010. A stroke diagnosis included intracerebral hemorrhage, cerebral infarction, and unspecified stroke in accordance with the Danish version of the International Classification of Diseases 10^th^ edition (ICD-10: I61, I63, I64). The patients were identified from the Danish Stroke Registry (DSR) [20], [21]. The DSR contains detailed data on the characteristics of all patients admitted with acute stroke to Danish hospitals from 2003 and onwards. Participation is mandatory for all hospitals in Denmark treating patients with acute stroke. This study was based on individual record linkage of data between Danish health care registries. Records can be linked across the primary and secondary Danish health care sectors and with data for most prescription drugs. Health-related data may be linked at the level of the individual who is assigned a unique 10-digit personal identification code upon birth, and Danish citizens have no disincentives to seek health care services which are largely/fully tax-financed.

### Use of antipsychotics

Exposure was defined as redemption of a prescription for antipsychotic medication before admission with stroke. Information was obtained from the Danish National Prescription Registry [22], which contains data from 1995 and onwards on all prescription drugs dispensed at Danish pharmacies. In Denmark, antipsychotics are available by prescription only, but antipsychotics used in hospitals are not registered in the Danish National Prescription Registry. We examined prescriptions in the antipsychotic drugs group (Anatomical Therapeutic Chemical (ATC) classification: N05A) [23] that had been redeemed before the date of the stroke. Included were both typical and atypical antipsychotics; antiemetics and lithium salts were excluded. Patients who filled a prescription within 90 days of the stroke were considered current users at the time of admission. Former users included patients who had filled a prescription for antipsychotics more than 90 days before their admission to hospital. Recent users were a subgroup of the latter group, namely those who had filled a prescription for antipsychotics 90 to 365 days before their admission for stroke. Never users included patients who had not filled a prescription for antipsychotics at any time prior to their hospital admission from 1995 and onwards.

### Clinical outcomes

#### Severity of stroke

The DSR holds information on the severity of the stroke at the time of admission. Severity is graded according to the Scandinavian Stroke Scale [20], [24], [25], which is a validated neurological scale that is widely used in Scandinavia. The scale evaluates level of consciousness; eye movement; power in the arm, hand, and leg; orientation; aphasia; facial paresis; and gait. The total score may range from 0 to 58 [25]. Stroke was classified as very severe (≤14), severe (15–29), moderate (30–44), and mild (≥45 pts). For reporting purposes, stroke severity was dichotomized into severe and very severe, and mild and moderate (0–29 pts versus 30–58 pts).

#### Length of hospital stay

Length of stay was defined as the time span from admission to discharge. The admission date was defined as the date the patient was admitted to the hospital with stroke or the date of stroke occurrence if the patient was already hospitalized with another diagnosis.

#### Mortality

30-day and one-year mortality was assessed using information from the Danish Civil Registration System [26], which maintains electronic records of changes in citizens’ vital status.

### Covariates

We retrieved data from the DSR and the Danish National Patient Registry [27] to adjust for comorbidity and risk factors. The Danish National Patient Registry was established in 1977 and keeps data concerning both admissions and discharges of patients, including up to 20 discharge diagnoses, and all surgical procedures performed in Danish hospitals. These data were used to calculate a Charlson comorbidity index score [28]. This index includes 19 disease categories, and we defined three groups according to level of comorbidity; no comorbidities, one comorbidity (moderate), and 2 or more comorbidities (severe). Cerebrovascular diseases were excluded from our comorbidity index, because former strokes and other neurological disorders were analyzed separately. The DSR contains information about stroke patients’ alcohol and tobacco use, and indicators of in-hospital care quality. The Danish IDA database [29] contains detailed data on socioeconomic factors including education, income, and employment status on the entire Danish population.

### Statistics

The clinical outcomes including stroke severity, length of stay, and 30-day mortality were compared for current versus former and never users of antipsychotic drugs.

#### Stroke severity

Dichotomized data on stroke severity were analyzed using logistic regression analysis. The results were reported as odds ratios (ORs). Covariates included in the analyses were: age group (five-year increments), gender, calendar period, modified Charlson’s index by comorbidity group, preadmission use of other drugs (including antihypertensive drugs, lipid-lowering drugs, antithrombotic drugs, and antidiabetic drugs), educational level, and former stroke. Educational level was used as an indicator of socioeconomic status. Data were stratified by gender.

#### Length of stay

Length of stay was analyzed using survival analysis with competing risks (death versus discharge) within 30 days of admission. We used the Aalen-Johansen method to estimate the probability (cumulative incidence) of discharge and death. The more often used Kaplan-Meier product-limit estimator corresponds to the hypothetical situation where deceased individuals are censored, whereas Aalen-Johansen curves estimate the probability of discharge by taking competing death into account [30]. Aalen-Johansen survival curves were obtained using the SAS macro presented in a paper by Rosthoj [31].

#### Mortality

Mortality was analyzed using survival analyses. Poisson regression with the GENMOD procedure in SAS version 9.2 (SAS Institute Inc, Cary, NC) was used for calculation of mortality rate ratios (MRRs). This method approximates a Cox regression. Covariates included are the same as in the analysis of stroke severity. Data were stratified by gender and age (over/under 70 years) in cumulative mortality curves. The age of 70 years was chosen for stratification to minimize the influence of dementia on our estimates. The prevalence of dementia in Western Europe was reported to be 2.6% for those aged 65 to 69 years, but doubled with every five-year increment in age [32]. In adjusted models, data were stratified by gender.

No data on the main outcome variables of antipsychotic medication use were missing. Data on at least one of the confounding variables including alcohol intake, smoking status, and Body Mass Index (BMI) were missing in 48.9% of the patients. Complete-case analyses were performed to evaluate the impact of missing data. We evaluated the robustness of the study findings by repeating the analyses after stratifying for age, gender, type of stroke, stroke severity, type of antipsychotics, former stroke, former neurological disorders, anti-dementia medication use and diabetes diagnoses. We adjusted for percentage of fulfilled indicators of in-hospital care quality including early admission to a specialized stroke unit, use of antiplatelet or anticoagulant therapy (ischemic stroke), early CT or MRI examination, assessment by a physiotherapist and an occupational therapist, and assessment of nutritional risk. In sub-analyses, recurrent users of antipsychotics were defined as current and former users who had filled three or more prescriptions, and they were compared with never users. Recent users were compared with current, former and never users.

We also did a supplementary analysis based on propensity score matching to reduce the risk of confounding by indication (Caliper method with 0.2 standard deviation of the logit of the estimated propensity score). In this analysis, current users of antipsychotics were matched with never users; we allowed up to five non-users for each antipsychotic user. We matched users on the following covariates: gender, age, former stroke, use of other drugs before admission (including antihypertensive drugs, lipid-lowering drugs, antithrombotic drugs and antidiabetic drugs), comorbidity index, and education level. We performed matching analysis, stratifying the propensity score in seven groups, with increasing propensity score. The matching was followed by regression analysis.

### Research ethics

The project was implemented as a register-based study based on anonymous data located at the Danish *Statistics Denmark* and was approved by the Danish Data Protection Agency. Register-based studies on anonymous data need no approval from the local clinical ethics committee in Denmark.

## Results

During the eight-year study period, we identified 81,143 patients who had been admitted to hospital for stroke. Among these, 2,770 (3.4%) were current users and 6,121 (7.5%) were former users of antipsychotic medication at the time of their admission (**Table 1**).

**Table 1:** Characteristics of acute stroke patients (n = 81,143) according to preadmission antipsychotic medication use.

Characteristics		Current antipsychotics users (n = 2,770, 3.41%)	Former antipsychotics users (n = 6,121, 7.54%)	Never antipsychotics users (n = 72,252, 89.0%)
Age	Mean (SD)	71.77 (13.44)	72.40 (13.59)	71.99 (13.28)
Age, distribution, n (%)	<70 years	1,148 (41.44)	2,388 (39.01)	29,012 (40.15)
	≥70 years	1,622 (58.56)	3,733 (60.99)	43,240 (59.85)
Gender, n (%)	Male	1,107 (39.96)	2,519 (41.15)	38,777 (53.67)
	Female	1,663 (60.04)	3,602 (58.85)	33,475 (46.33)
Severity of stroke, n (%)	Mild (≥45 pts)	1,095 (39.53)	2,799 (45.73)	37,489 (51.89)
	Moderate (30–44 pts)	502 (18.12)	981 (16.03)	10,352 (14.33)
	Severe (15–29 pts)	307 (11.08)	605 (9.88)	6,217 (8.60)
	Very severe (≤14 pts)	380 (13.72)	685 (11.19)	6,825 (9.45)
	Unknown	486 (17.55)	1,051 (17.17)	11,369 (15.74)
Type of stroke, n (%)	Intracerebral hemorrhage	313 (11.30)	724 (11.83)	8,067 (11.17)
	Ischemic	1,906 (68.81)	4,245 (69.35)	51,588 (71.40)
	Unspecified	551 (19.89)	1,152 (18.82)	12,597 (17.43)
Comorbidity index, n (%)*	No comorbidity, 0	1,108 (40.00)	2,476 (40.45)	37,401 (51.76)
	Moderate comorbidity, 1	626 (22.60)	1,289 (21.06)	12,123 (16.78)
	Severe comorbidity, 2+	1,036 (37.40)	2,356 (38.49)	22,728 (31.46)
Former stroke, n (%)	Yes	717 (25.88)	1,480 (24.18)	13,772 (19.06)
	No	1,803 (65.09)	4,226 (69.04)	54,166 (74.97)
	Missing	250 (9.03)	415 (6.78)	4,314 (5.97)
Education, n (%)	Long	49 (1.77)	125 (2.04)	2,015 (2.79)
	Medium	184 (6.64)	422 (6.89)	6,414 (8.88)
	Short	605 (21.84)	1,509 (24.65)	20,596 (28.51)
	High school	46 (1.66)	71 (1.16)	1,038 (1.44)
	Primary school	1,397 (50.43)	2,939 (48.02)	30,368 (42.03)
	Missing	489 (17.65)	1,055 (17.24)	11,821 (16.36)
Type of antipsychotics†	Typical	1,635 (20.80)	6,230 (79.21)	0.00 (0.00)
	Atypical	1,323 (58.80)	927 (41.20)	0.00 (0.00)
Preadmission drug use	Antihypertensive drug, n (%)	2,119 (76.50)	4,871 (79.58)	50,178 (69.45)
	Lipid-lowering drugs, n (%)	572 (20.65)	1,562 (25.52)	17,135 (23.72)
	Platelet inhibitors, n (%)	1,556 (56.17)	3,663 (59.84)	36,547 (50.58)
	Antidiabetic drugs, n (%)	389 (14.04)	850 (13.89)	8,274 (11.45)
BMI, n (%)	<18.5	101 (3.65)	219 (3.58)	2,149 (2.97)
	18.5-<25	663 (23.94)	1,705 (27.85)	20,365 (28.19)
	≥25	822 (29.68)	1,796 (29.34)	23,302 (32.25)
	Missing	1,184 (42.74)	2,401 (39.23)	26,436 (36.59)
Alcohol units/week, n (%)	≤14 for women/≤ 21 for men	1,842 (66.50)	4,364 (71.30)	55,108 (76.27)
	>14 for women/> 21 for men	208 (7.51)	507 (8.28)	5,068 (7.01)
	Missing	720 (25.99)	1,250 (20.42)	12,076 (16.71)
Smoking habits, n (%)	Never	551 (19.89)	1,528 (24.96)	20,989 (29.05)
	Daily or occasionally	1,069 (38.59)	2,183 (35.66)	23,158 (32.05)
	Former (>½ year)	356 (12.85)	1,005 (16.42)	14,410 (19.94)
	Missing	794 (28.66)	1,405 (22.95)	13,695 (18.95)

Modified Charlson’s index (cerebrovascular disorders excluded).

Sums of proportions can exceed total number of users; some patients will appear in both categories, e.g. current use of atypical and former use of typical antipsychotics.

### Stroke severity

Data on stroke severity were available for 68,237 (84.7%) patients of whom 15,019 (22.0%) had severe or very severe stroke (**Table 1**). The adjusted ORs for the risk of having a severe or very severe stroke were higher for current users (1.43; 95% confidence interval [CI] 1.29–1.58) and former users (1.12; 95% CI, 1.05–1.21) of antipsychotics compared to never users (**Table 2**).

**Table 2:** Association between preadmission antipsychotic medication use and risk of severe and very severe stroke on the Scandinavian Stroke Scale on admission for acute stroke.

Antipsychotic use		Number of users (%)	Severe and very severe stroke. Odds ratios* (95% Wald CI)
Overall	Current users	2,284 (3.35)	1.43 (1.29–1.58)
	Former users	5,070 (7.43)	1.12 (1.05–1.21)
	Never users	60,879 (89.2)	1.00 (reference)
Female	Current users	1,364 (2.00)	1.26 (1.11–1.43)
	Former users	2,961 (4.34)	1.11 (1.01–1.21)
	Never users	27,902 (40.9)	1.00 (reference)
Male	Current users	920 (1.35)	1.76 (1.50–2.05)
	Former users	2,109 (3.09)	1.13 (1.01–1.27)
	Never users	32,977 (48.3)	1.00 (reference)

Stroke severity on the Scandinavian Stroke Scale dichotomized into two groups (mild and moderate versus severe and very severe). Adjusted for former stroke, pre-stroke drug use (lipid-lowering drugs, antihypertensive drugs, antidiabetic drugs, platelet inhibitors), education level, age group, year of admission, and modified Charlson’s index (cerebrovascular disorders excluded). Overall estimates adjusted for gender. Interaction between genders (*p*<0.01).

### Length of stay

Preadmission users of antipsychotics were less likely to be discharged from hospital within 30 days of their admission for stroke than never users (probability of non-discharge, 27.0% versus 21.9%). Current users were less likely to be discharged than former users (**Supporting Table S1 in File S1**).

### Mortality

**Figure 1:**
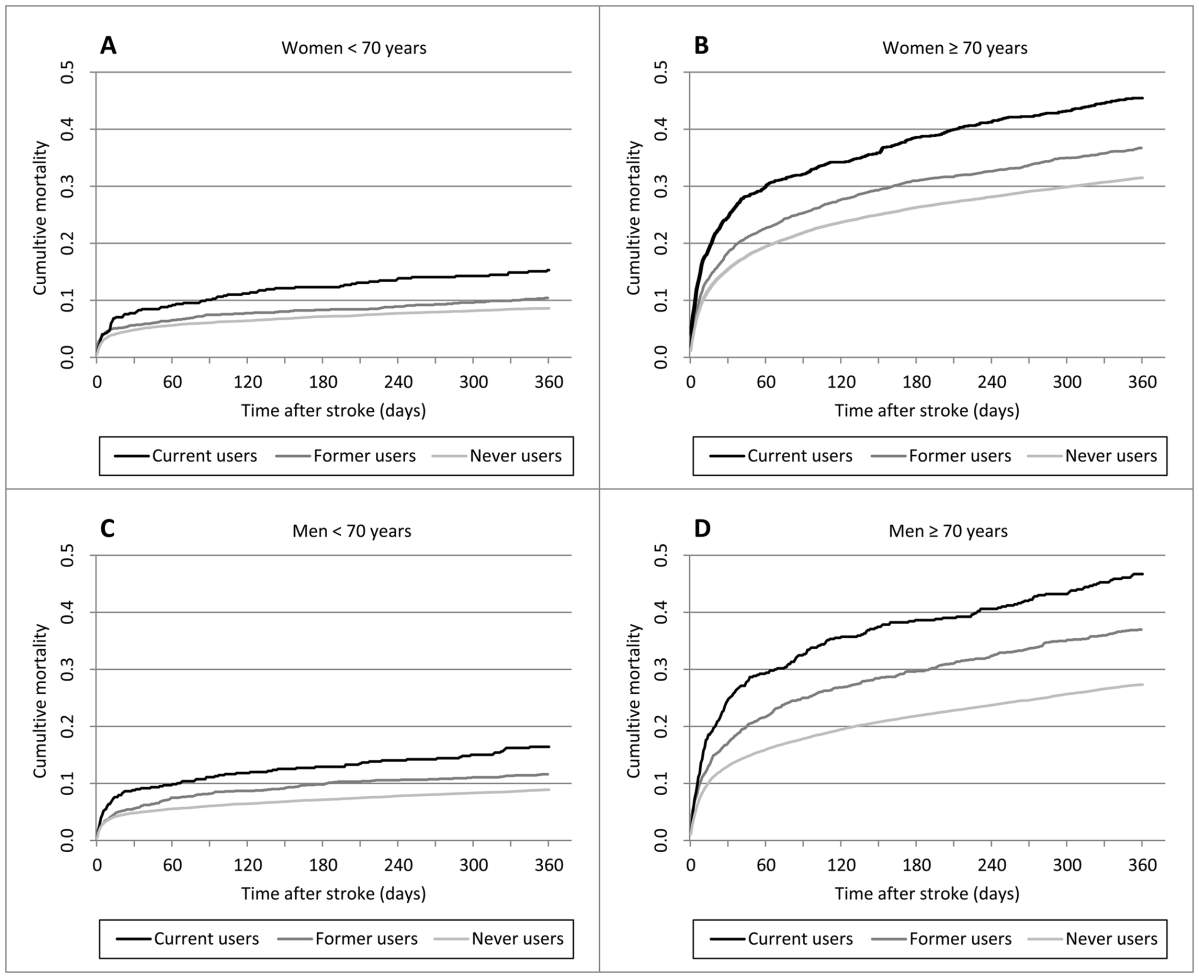
Association between preadmission antipsychotic medication and cumulative 1-year post-stroke mortality stratified by gender and age.

The overall cumulative mortality was 10.8% within 30 days and 22.1% within one year after the stroke. In the crude analysis, use of antipsychotics was associated with an increased 30-day post-stroke mortality (MRRs, current users 1.83; 95% CI, 1.67–2.01; former users 1.22; 95% CI, 1.13–1.31) compared with never users. Data was stratified by gender and age (over/under 70 years) (**Figure 1**). Adjustment for potential confounders (type of stroke, former stroke, pre-stroke drug, and education level) attenuated the 30-day MRRs for current and former users of antipsychotics, but the associations remained statistically significant. However, when stroke severity and comorbidity were included in the model, only current users had an increased mortality compared with never users (adjusted MRRs, current users 1.42; 95% CI, 1.29–1.55; former users 1.05; 95% CI, 0.98–1.14) (**Table 3**).

**Table 3:** Association between preadmission antipsychotic medication use and 30-day mortality in acute stroke patients in various adjusted models.

			Adjusted mortality rate ratios (95% CI)		
Antipsychotic use		Number of users (%)	Model 1*	Model 2†	Model 3‡
Overall	Current users	490 (5.58)	1.83 (1.67–2.01)	1.64 (1.50–1.80)	1.42 (1.29–1.55)
	Former users	787 (8.97)	1.22 (1.13–1.31)	1.12 (1.04–1.21)	1.05 (0.98–1.14)
	Never users	7,498 (85.4)	1.00 (reference)	1.00 (reference)	1.00 (reference)
Female	Current users	310 (3.53)	1.74 (1.55–1.96)	1.52 (1.35–1.70)	1.38 (1.23–1.56)
	Former users	511 (5.82)	1.20 (1.09–1.31)	1.12 (1.02–1.23)	1.06 (0.97–1.16)
	Never users	4,002 (45.6)	1.00 (reference)	1.00 (reference)	1.00 (reference)
Male	Current users	180 (2.05)	2.00 (1.72–2.33)	1.93 (1.66–2.25)	1.49 (1.28–1.74)
	Former users	276 (3.15)	1.24 (1.10–1.40)	1.11 (0.98–1.25)	1.04 (0.92–1.18)
	Never users	3,496 (39.8)	1.00 (reference)	1.00 (reference)	1.00 (reference)

Model 1: adjusted for age group and calendar period. Overall estimates adjusted for gender in all models.

†Model 2: further adjusted for type of stroke, former stroke, pre-stroke drug use (lipid-lowering drugs, antihypertensive drugs, antidiabetic drugs, platelet inhibitors) and education level.

‡Model 3: further adjusted for year of admission, severity of stroke, and modified Charlson’s index (cerebrovascular disorders excluded). No interaction between genders in model 3 (*p* = 0.79).

The MRRs remained virtually the same when we stratified by age (over/under 70 years), stroke type (intracerebral hemorrhage, cerebral infarction, or unspecified stroke), stroke severity, former stroke, former neurological disorders and diabetes (**Supporting Tables S2–S6 in File S1**). Anti-dementia medication use acted as a proxy for dementia. Use of anti-dementia medication was associated with increased post-stroke mortality, but when we excluded this group, the MRRs were virtually the same as in our main analysis (**Supporting Table S7 in File S1**). Current and former users of atypical antipsychotics tended to have an increased mortality compared to typical antipsychotics users (**Supporting Table S8 in File S1**). Former users who filled their latest prescription from 4 to 12 months before their stroke (recent users) did not have a significantly higher mortality than those who filled their last prescription more than 12 months ago (**Table 4**). No systematic differences from the main analysis were seen if we defined recurrent users as those who had filled three or more prescriptions for antipsychotics (**Supporting Table S9 in File S1**). Adjusting for indicators of in-hospital care quality showed no changes in our estimates, but should be interpreted with caution due to a significantly higher mortality in the missing group (**Supporting Table S10 in File S1**). Complete-case analyses, including smoking and alcohol use, and our analysis based on propensity score matching did not significantly alter the estimates (**Supporting Table S11–S12 in File S1**).

**Table 4:** Association between preadmission antipsychotic medication use and 30-day mortality in acute stroke patients according to prescription period.

Prescription period (months before admission for stroke)		Mortality rate ratio* (95% CI)
Overall	Current (0–3 months)	1.41 (1.28–1.54)
	Recent (4–12 months)	1.16 (0.98–1.38)
	Former (12+ months)	1.03 (0.95–1.12)
	Never	1.00 (reference)

Adjusted for gender, former stroke, pre-stroke drug use (lipid-lowering drugs, antihypertensive drugs, antidiabetic drugs, platelet inhibitors), education level, age group, year of admission, and modified Charlson’s index (cerebrovascular disorders excluded).

## Discussion

This large population-based cohort study showed that post-stroke mortality was higher in current users of antipsychotics than in former and never users of antipsychotics. The risk of severe stroke was higher in current and former users than in never users.

Neuronal protection as a therapeutic target in acute stroke has been intensively investigated, and it has been proposed that antipsychotic medication may have a neuroprotective potential [1]. The biological mechanism underlying this effect is not clear, but glutamate-mediated excitotoxicity seems to contribute to the cerebral damage in stroke; i.e. the release of neurotransmitters during stroke has been found to aggravate the neuronal damage in ischemic tissue [6]–[9]. The antiserotonergic and antidopaminergic effects of antipsychotics are believed to reduce the release of neurotransmitters, and this will decrease the excitotoxic effect of glutamate which may, in turn, protect the neurons [10]. However, animal studies are ambiguous; dopamine receptor antagonists may impair recovery after brain damage in rats [11]–[13], although recent studies have found that focal ischemic brain damage was reduced in mice if various antipsychotics were given while ischemia was being induced [2]–[5].

The hypothesis of neuroprotection from antipsychotics in stroke was addressed in a population-based cohort study from Taiwan [1]. This study included 285 in-patients with schizophrenia and 2,425 sex, age, and stroke type-matched patients without schizophrenia. Patients with schizophrenia had a lower 90-day post-stroke mortality (hazard ratio [HR], 0.35; 95% CI, 0.21–0.57) than those with no history of schizophrenia, but the effect was restricted to those who used antipsychotic medication within three months prior to their stroke (HR, 0.18; 95% CI, 0.09–0.37).

The study was, however, challenged by another Taiwanese study which found an increased post-stroke all-cause five-year mortality in both in-patients and out-patients with schizophrenia (HR, 1.23; 95% CI, 1.06–1.41) [33], and by a Canadian study showing an increased 28-days post-stroke mortality in patients with schizophrenia and related psychosis (OR, 1.41; 95% CI, 1.00–1.98) [34]. However, neither of these studies included data on antipsychotic medication. In a British study, patients with severe mental illnesses, including schizophrenia, had increased stroke death rates compared with the background population, and the HRs decreased with age. Prescription of antipsychotic medication did not modify the results [35]. In an American study of various drugs thought to be detrimental to stroke recovery (based on animal studies, including antipsychotics [11]–[13]), post-stroke recovery was impaired in the treatment group compared with a group receiving drugs not believed to impair post-stroke recovery (“neutral” drugs) [18]. Albeit its study population was small, this American study gave rise to advice not to use antipsychotics in patients who had suffered a stroke [19].

Antipsychotics are used for several conditions including schizophrenia and other psychotic disorders, bipolar affective disorder, unipolar depression, personality disorders, post-traumatic stress disorder, obsessive compulsive disorder, Tourette's syndrome, attention deficit hyperactivity disorder, and agitation in dementia [14], [15]. In schizophrenia, they play an important role by relieving psychotic symptoms like delusions and hallucinations. A meta-analysis of randomized controlled trials showed that antipsychotics used in schizophrenia reduced relapse rates and hospitalizations and improved quality of life compared with placebo, but also that their use was associated with adverse effects. No significant effect on suicide attempts and mortality was found, but the data were sparse and follow-up was short [36]. A Finnish nationwide study with 11-years of follow-up found that antipsychotics reduced overall mortality (adjusted HR, 0.68; 95% CI, 0.65–0.71) [16], and other studies showed similar results in those who were treated with multiple and atypical antipsychotics [16], [37]–[40]. Conversely, the use of antipsychotics against agitation in patients with dementia seems to increase all-cause mortality by 20–60% [17].

The strengths of the present study spring from its use of a large nationwide population-based cohort with individual-based data linkage and a validated stroke database with prospectively collected data that also cover stroke severity [21]. We followed all patients treated for stroke in any Danish hospital from 2003–2010 and had complete follow-up on mortality. Bias due to selection of study participants is therefore an unlikely explanation for our findings. However, stroke causing death before hospital admission was not registered in the database. Psychiatric patients may have longer delay before seeing healthcare professionals and a higher pre-hospital mortality than non-psychiatric patients [34]. However, we have underestimated the association if there is a tendency for the database to contain data on milder rather than more severe cases of stroke in persons who use antipsychotics. To our knowledge, no other study has addressed the association between antipsychotics and stroke severity.

The registration of death in Denmark is complete and valid, wherefore we expect our post-stroke mortality estimates to be very accurate. The stroke diagnoses and the severity assessments were based on the clinical and paraclinical examinations made by the physician in charge of the stroke treatment. However, users of antipsychotics, including patients with various degrees of psychosis and dementia, may be more difficult to examine, diagnose, and treat than other patients, which may cause some of the data in the register to be missing or imprecise. Stroke severity was assessed on admission, but follow-up data on long-term disability were not available. Data on medication were based on prescription fillings, but no data were available on patient compliance, the exact doses or indications for drug use. Doses may vary depending on the indication. In-patients in psychiatric wards are treated with antipsychotics in the hospital and therefore do not redeem prescriptions. Even some out-patients receive long-acting injections of antipsychotics at a hospital center. This may lead to an underestimation of the association, but the number of such patients is thought to be very small in the present study.

We adjusted for several important confounders such as age, gender, socioeconomic factors, pre-stroke medication use, and comorbidity by stratification and adjustment in multivariate regression analyses. The association between antipsychotics and mortality is not necessarily due to an adverse effect of the drug; it could be confounded by the treatment indication. This is an important limitation of non-randomized studies; thus, we do not know what the risk would have been in current users of antipsychotics had they not taken these drugs. In our study, they tended to have a higher risk of death and to suffer more severe stroke than persons who had previously fulfilled the indication for treatment (former users, including recent users). These findings provide some evidence against a strong effect of confounding by indication as former users may share some genetic and environmental risk factors with current users. However, the argument is not a strong one as treatment is often discontinued for good reasons; former users may have less severe symptoms at the time of the stroke. Unfortunately, we had no information about the indication for the treatment, but the indication is more likely to be schizophrenia in the younger stroke patients and dementia in the older. Dementia was thought to be an important confounder. Still, our estimates were not affected by age stratification and exclusion of patients with dementia, indicating that the higher mortality in antipsychotics users was not driven primarily by dementia. As the use of antipsychotics over a long period of time indicates more chronic conditions, we conducted a sub-analysis using redemption of three or more antipsychotics prescriptions to identify the exposed group, but our findings were the same for both former and current users. The quality of treatment in the acute phase of the stroke could influence on the long-term outcomes. However, the quality indicators alone should be interpreted with caution as it may not be clinical relevant to apply all treatment to all patients, e.g. it is not relevant with physiotherapy to a moribund stroke patient.

Metabolic syndrome is always a concern in patients using antipsychotic medication, and we had incomplete information on risk factors such as smoking. We adjusted for preadmission drugs used in treatment of e.g. hypertension and diabetes. Furthermore, stratification on diabetes and our complete-case analyses showed no differences in mortality from our main analysis, indicating that the excess mortality in current antipsychotics users is not mainly driven by diabetes, smoking and alcohol use. This is in accordance with another Danish study that showed no significant increase in 30-days post-stroke mortality with a diagnosis of diabetes or alcohol/tobacco use [41]. Our BMI data were too sparse to be evaluated, but several studies report a paradox lower post-stroke mortality in overweight patients compared to normal weight patients, so high BMI alone does not seem to contribute to an increased risk of post-stroke death [42], [43]. Our stratifications and additional sub-analyses, including propensity score matching, did not affect the overall MRRs associated with antipsychotics, but we cannot entirely exclude residual confounding by factors for which we did not account. Randomized controlled trials are needed to address this thoroughly. The neuroprotective effect may therefore theoretically have been present, but could be counteracted by metabolic adverse effects from the antipsychotics or other factors associated with the use of antipsychotics.

## Summary

Preadmission use of antipsychotics was associated with a higher risk of severe stroke, a longer duration of hospital stay, and a higher post-stroke mortality, even after adjustment for known confounders. Antipsychotics play an important role in the treatment of many psychiatric conditions, but our findings do not support that they reduce the severity of the stroke or the post-stroke mortality.

## Supporting Information

File S1 **File includes Supporting Tables S1–S12.** Supporting Table S1: Association between preadmission antipsychotic medication use and length of hospital stay in a competing risk set-up. Supporting Table S2: Association between preadmission antipsychotic medication use and 30-day mortality in acute stroke stratified by age. Supporting Table S3: Association between preadmission antipsychotic medication use and 30-day mortality in acute stroke stratified by type of stroke and stroke severity on the Scandinavian Stroke Scale. Supporting Table S4: Association between preadmission antipsychotic medication use and 30-day mortality stratified by former stroke. Supporting Table S5: Association between preadmission antipsychotic medication use and 30-day mortality stratified by former neurological disorder. Supporting Table S6: Association between preadmission antipsychotic medication use and 30-day mortality stratified by a diagnosis of diabetes. Supporting Table S7: Association between preadmission antipsychotic medication use and 30-day mortality stratified by preadmission anti-dementia medication use. Supporting Table S8: Association between preadmission antipsychotic medication use and 30-day mortality in acute stroke according to typical versus atypical antipsychotics use. Supporting Table S9: Association between preadmission antipsychotic medication use and 30-day mortality in users having filled three or more prescriptions before admission for acute stroke. Supporting Table S10: Association between quality of in-hospital care, preadmission antipsychotic medication use and 30-day mortality. Supporting Table S11: Association between antipsychotic medication use and 30-day mortality in complete-case analyses of alcohol and tobacco use. Supporting Table S12: Association between preadmission antipsychotic medication use and 30-day mortality. Propensity score matching analysis.
(PDF)
