# Supplementary material for: Post-Stroke Mortality, Stroke Severity, and Preadmission Antipsychotic Medicine Use – A Population-Based Cohort Study
Source: PLoS One. 2014 Jan 8;9(1):e84103. doi: 10.1371/journal.pone.0084103 (PMC3885530; doi:10.1371/journal.pone.0084103)
Supplement: File S1 — File includes Supporting Tables S1–S12. Supporting Table S1: Association between preadmission antipsychotic medication use and length of hospital stay in a competing risk set-up. Supporting Table S2: Association between preadmission antipsychotic medication use and 30-day mortality in acute stroke stratified by age. Supporting Table S3: Association between preadmission antipsychotic medication use and 30-day mortality in acute stroke stratified by type of stroke and stroke severity on the Scandinavian Stroke Scale. Supporting Table S4: Association between preadmission antipsychotic medication use and 30-day mortality stratified by former stroke. Supporting Table S5: Association between preadmission antipsychotic medication use and 30-day mortality stratified by former neurological disorder. Supporting Table S6: Association between preadmission antipsychotic medication use and 30-day mortality stratified by a diagnosis of diabetes. Supporting Table S7: Association between preadmission antipsychotic medication use and 30-day mortality stratified by preadmission anti-dementia medication use. Supporting Table S8: Association between preadmission antipsychotic medication use and 30-day mortality in acute stroke according to typical versus atypical antipsychotics use. Supporting Table S9: Association between preadmission antipsychotic medication use and 30-day mortality in users having filled three or more prescriptions before admission for acute stroke. Supporting Table S10: Association between quality of in-hospital care, preadmission antipsychotic medication use and 30-day mortality. Supporting Table S11: Association between antipsychotic medication use and 30-day mortality in complete-case analyses of alcohol and tobacco use. Supporting Table S12: Association between preadmission antipsychotic medication use and 30-day mortality. Propensity score matching analysis. (PDF) [file pone.0084103.s001.pdf]

ONLINE SUPPORTING INFORMATION

Post-stroke mortality, stroke severity, and preadmission antipsychotic medicine use – a population-based cohort study

Anders Prior[1,2]; Thomas Munk Laursen[3]; Karen Kjær Larsen[1,2]; Søren Paaske Johnsen[4]; Jakob Christensen[5]; Grethe Andersen[5]; Mogens Vestergaard[1,2]

[1]Research Unit for General Practice, Department of Public Health, Aarhus University, Denmark;

[2]Section for General Practice, Department of Public Health, Aarhus University, Denmark;

[3]National Centre for Register-based Research, Aarhus University, Denmark;

[4]Department of Clinical Epidemiology, Aarhus University Hospital, Denmark;

[5]Department of Neurology, Aarhus University Hospital, Denmark

Supporting Table S1. Association between preadmission antipsychotic medication use and length of hospital stay in a competing risk set-up

Antipsychotics use	Probability of non-discharge within 30 days (%)*
Overall	
Current users	27.0
Former users	24.4
Never users	21.9
Female	
Current users	28.1
Former users	25.8
Never users	24.2
Male	
Current users	25.4
Former users	22.5
Never users	20.0

* Unadjusted analysis.

Supporting Table S2. Association between preadmission antipsychotic medication use and 30-day mortality in acute stroke stratified by age

Antipsychotics use	Mortality Rate Ratios*(95% CI)
< 70 years	
Current users	1.42 (1.29-1.58)
Former users	1.07 (0.99-1.16)
Never users	1.00 (reference)
≥ 70 years	
Current users	1.44 (1.17-1.77)
Former users	0.96 (0.80-1.15)
Never users	1.00 (reference)

* Adjusted for gender, former stroke, pre-stroke drug use (lipid-lowering drugs, antihypertensive drugs, antidiabetic drugs, platelet inhibitors), education level, age within age group, year of admission, and modified Charlson's index (cerebrovascular disorders excluded).

Supporting Table S3. Association between preadmission antipsychotic medication use and 30-day mortality in acute stroke stratified by type of stroke and stroke severity on the Scandinavian Stroke Scale

	Type of stroke Mortality rate ratios* (95% CI)			Severity Mortality rate ratios* (95% CI)	
Antipsychotics use	Intracerebral hemorrhage (ICD-10: I61)	Cerebral infarction (ICD-10: I63)	Unspecified stroke (ICD-10: I64)	Mild and moderate stroke	Severe and very severe stroke
Overall					
Current users	1.28 (1.08-1.51)	1.51 (1.33-1.72)	1.43 (1.16-1.77)	1.34 (1.06-1.69)	1.38 (1.23-1.55)
Former users	1.01 (0.88-1.15)	1.12 (1.01-1.24)	0.97 (0.81-1.16)	0.89 (0.74-1.08)	1.07 (0.97-1.17)
Never users	1.00 (reference)	1.00 (reference)	1.00 (reference)	1.00 (reference)	1.00 (reference)
Female					
Current users	1.19 (0.97-1.46)	1.52 (1.29-1.78)	1.43 (1.08-1.89)	1.11 (0.81-1.53)	1.36 (1.18-1.57)
Former users	1.03 (0.88-1.22)	1.08 (0.95-1.23)	1.05 (0.85-1.31)	0.83 (0.64-1.08)	1.09 (0.97-1.22)
Never users	1.00 (reference)	1.00 (reference)	1.00 (reference)	1.00 (reference)	1.00 (reference)
Male					
Current users	1.56 (1.18-2.07)	1.48 (1.19-1.83)	1.44 (1.04-1.98)	1.62 (1.16-2.27)	1.47 (1.21-1.77)
Former users	0.97 (0.78-1.20)	1.19 (1.00-1.41)	0.82 (0.59-1.13)	0.95 (0.72-1.25)	1.02 (0.87-1.20)
Never users	1.00 (reference)	1.00 (reference)	1 .00 (reference)	1.00 (reference)	1.00 (reference)

* Adjusted for former stroke, pre-stroke drug use (lipid-lowering drugs, antihypertensive drugs, antidiabetic drugs, platelet inhibitors), education level, age group, year of admission, and modified Charlson's index (cerebrovascular disorders excluded). Overall estimates adjusted for gender.

Supporting Table S4. Association between preadmission antipsychotic medication use and 30-day mortality stratified by former stroke

Former stroke	Mortality rate ratios*(95% CI)
Yes	
Current users of antipsychotics	1.44 (1.21-1.71)
Former users of antipsychotics	1.09 (0.94-1.26)
Never users of antipsychotics	1.00 (reference)
No	
Current users of antipsychotics	1.54 (1.36-1.74)
Former users of antipsychotics	1.04 (0.94-1.14)
Never users of antipsychotics	1.00 (reference)
Unknown	
Current users of antipsychotics	1.10 (0.88-1.38)
Former users of antipsychotics	1.06 (0.87-1.28)
Never users of antipsychotics	1.00 (reference)

* Adjusted for gender, pre-stroke drug use (lipid-lowering drugs, antihypertensive drugs, antidiabetic drugs, platelet inhibitors), education level, age group, year of admission, and modified Charlson's index (cerebrovascular disorders excluded).

Supporting Table S5. Association between preadmission antipsychotic medication use and 30-day mortality stratified by former neurological disorder

Former neurological disorders†	Mortality rate ratios*(95% CI)
Yes	
Current users of antipsychotics	1.35 (1.16-1.56)
Former users of antipsychotics	1.02 (0.91-1.15)
Never users of antipsychotics	1.00 (reference)
No	
Current users of antipsychotics	1.46 (1.30-1.64)
Former users of antipsychotics	1.08 (0.98-1.18)
Never users of antipsychotics	1.00 (reference)

* Adjusted for gender, former stroke, pre-stroke drug use (lipid-lowering drugs, antihypertensive drugs, antidiabetic drugs, platelet inhibitors), education level, age group, year of admission, and modified Charlson's index (cerebrovascular disorders excluded).

† Neurological and cerebrovascular disorders included (ICD-10): DG00-DG99, DI60-DI69.

Supporting Table S6. Association between preadmission antipsychotic medication use and 30-day mortality stratified by a diagnosis of diabetes

Diabetes†	Mortality rate ratios*(95% CI)
Yes	
Current users of antipsychotics	1.36 (1.05-1.75)
Former users of antipsychotics	1.19 (0.98-1.44)
Never users of antipsychotics	1.00 (reference)
No	
Current users of antipsychotics	1.43 (1.29-1.58)
Former users of antipsychotics	1.02 (0.94-1.11)
Never users of antipsychotics	1.00 (reference)

* Adjusted for gender, former stroke, pre-stroke drug use (lipid-lowering drugs, antihypertensive drugs, antidiabetic drugs, platelet inhibitors), education level, age group, year of admission, and modified Charlson's index (cerebrovascular disorders and diabetes excluded).

† Diabetes diagnoses (ICD-10): E10.0, E10.1, E10.9, E11.0, E11.1, E11.9

Supporting Table S7. Association between preadmission antipsychotic medication use and 30-day mortality stratified by preadmission anti-dementia medication use

Anti-dementia medication use†	Mortality rate ratios*(95% CI)
Yes	
Current users of antipsychotics	1.75 (1.24-2.46)
Former users of antipsychotics	1.46 (1.06-2.01)
Never users of antipsychotics	1.00 (reference)
No	
Current users of antipsychotics	1.41 (1.28-1.55)
Former users of antipsychotics	1.04 (0.97-1.13)
Never users of antipsychotics	1.00 (reference)

* Adjusted for gender, former stroke, pre-stroke drug use (lipid-lowering drugs, antihypertensive drugs, antidiabetic drugs, platelet inhibitors), education level, age group, year of admission, and modified Charlson's index (cerebrovascular disorders excluded).

† Prescription on anti-dementia medication (ATC-groups: N06DA, N06DX) redeemed within 12 months before the admission with stroke.

Supporting Table S8. Association between preadmission antipsychotic medication use and 30-day mortality in acute stroke according to typical versus atypical antipsychotics use

	Mortality rate ratios* (95% CI)		
Antipsychotics use	Typical	Atypical	Both typical and atypical
Current users†	1.44 (1.25-1.66)	1.93 (1.65-2.26)	1.58 (1.03-2.42)
Former users†	1.06 (0.98-1.15)	1.52 (1.23-1.89)	1.55 (1.21-1.99)
Never users	1.00 (reference)	1.00 (reference)	1.00 (reference)

* Adjusted for gender, former stroke, pre-stroke drug use (lipid-lowering drugs, antihypertensive drugs, antidiabetic drugs, platelet inhibitors), education level, age group, year of admission, and modified Charlson's index (cerebrovascular disorders excluded).

† Current atypical and former typical use: MRR (95% CI) = 1.67 (1.37-2.04)
Current typical and former atypical use: MRR (95% CI) = 1.63 (0.98-2.71)

Supporting Table S9. Association between preadmission antipsychotic medication use and 30-day mortality in users having filled three or more prescriptions before admission for acute stroke

Antipsychotics use	Mortality rate ratios*(95% CI)
Current users	1.37 (1.24-1.51)
Former users	1.07 (0.97-1.18)
Never users	1.00 (reference)

* Adjusted for gender, former stroke, pre-stroke drug use (lipid-lowering drugs, antihypertensive drugs, antidiabetic drugs, platelet inhibitors), education level, age group, year of admission, and modified Charlson's index (cerebrovascular disorders excluded).

Supporting Table S10. Association between quality of in-hospital care, preadmission antipsychotic medication use and 30-day mortality

Quality of in-hospital care†	Mortality rate ratios*(95% CI)
Missing	5.98 (5.62-6.37)
0% - 19%	1.18 (1.08-1.30)
20% - 39%	1.11 (1.03-1.21)
40% - 59%	0.92 (0.85-1.00)
60% - 79%	1.08 (1.01-1.15)
80% - 100%	1.00 (reference)

Antipsychotics use	Mortality rate ratios‡ (95% CI)
Current users	1.31 (1.19-1.43)
Former users	1.02 (0.95-1.10)
Never users	1.00 (reference)

* Adjusted for gender, former stroke, pre-stroke drug use (lipid-lowering drugs, antihypertensive drugs, antidiabetic drugs, platelet inhibitors), education level, age group, year of admission, and modified Charlson's index (cerebrovascular disorders excluded).

† Percentage of fulfilled indicators of in-hospital care quality in the acute phase of the stroke.

‡ Adjusted for gender, former stroke, pre-stroke drug use (lipid-lowering drugs, antihypertensive drugs, antidiabetic drugs, platelet inhibitors), education level, age group, year of admission, modified Charlson's index (cerebrovascular disorders excluded), and quality of in-hospital care.

Supporting Table S11. Association between antipsychotic medication use and 30-day mortality in complete-case analyses of alcohol and tobacco use

Antipsychotics use	Mortality rate ratios*(95% CI)
Alcohol use†	
Current users of antipsychotics	1.44 (1.26-1.64)
Former users of antipsychotics	1.03 (0.93-1.13)
Never users of antipsychotics	1.00 (reference)
Tobacco use‡	
Current users of antipsychotics	1.53 (1.33-1.76)
Former users of antipsychotics	0.99 (0.89-1.10)
Never users of antipsychotics	1.00 (reference)

* Adjusted for gender, former stroke, pre-stroke drug use (lipid-lowering drugs, antihypertensive drugs, antidiabetic drugs, platelet inhibitors), education level, age group, year of admission, and modified Charlson's index (cerebrovascular disorders excluded).

† Adjusted for alcohol use, missings excluded.

‡ Adjusted for tobacco use, missings excluded.

Supporting Table S12. Association between preadmission antipsychotic medication use and 30-day mortality. Propensity score matching analysis

Propensity score	Mortality rate ratios*(95% CI)
2†	
Current users of antipsychotics	2.67 (1.10-6.47)
Former users of antipsychotics	1.48 (0.81-2.71)
Never users of antipsychotics	1.00 (reference)
5	
Current users of antipsychotics	1.72 (1.28-2.31)
Former users of antipsychotics	1.31 (1.05-1.64)
Never users of antipsychotics	1.00 (reference)
8	
Current users of antipsychotics	1.56 (1.27-1.91)
Former users of antipsychotics	1.13 (0.96-1.32)
Never users of antipsychotics	1.00 (reference)
11	
Current users of antipsychotics	1.64 (1.36-1.97)
Former users of antipsychotics	1.08 (0.92-1.26)
Never users of antipsychotics	1.00 (reference)
14	
Current users of antipsychotics	1.51 (1.24-1.84)
Former users of antipsychotics	1.09 (0.93-1.27)
Never users of antipsychotics	1.00 (reference)
17	
Current users of antipsychotics	1.63 (1.28-2.08)
Former users of antipsychotics	1.13 (0.93-1.39)
Never users of antipsychotics	1.00 (reference)
20	
Current users of antipsychotics	1.40 (1.03-1.90)
Former users of antipsychotics	1.02 (0.78-1.33)
Never users of antipsychotics	1.00 (reference)

* Adjusted for gender, former stroke, pre-stroke drug use (lipid-lowering drugs, antihypertensive drugs, antidiabetic drugs, platelet inhibitors), education level, age group, year of admission, and modified Charlson's index (cerebrovascular disorders excluded).

† Very few cases in this group.
